# Rhino-Orbital Cerebral Mucormycosis in a Non-diabetic Patient Following COVID-19 Infection: A Case Report and Literature Review

**DOI:** 10.7759/cureus.32884

**Published:** 2022-12-23

**Authors:** Maha Niazi, Isabella Vittorino, Hla Thwe, Benish Alam, Ayoola O Awosika, Adekunle E Omole

**Affiliations:** 1 Internal Medicine, Royal Alexandra Hospital, Edmonton, CAN; 2 Internal Medicine, Universidad Libre Barranquilla, Barranquilla, COL; 3 Internal Medicine, University of Medicine, Yangon, MMR; 4 Internal Medicine, Karachi Medical and Dental College, Karachi, PAK; 5 College of Medicine, The Ohio State University College of Medicine, Columbus, USA; 6 College of Health Sciences and Professions, Ohio University, Athens, USA; 7 Pathophysiology, American University of Antigua, Antigua, ATG; 8 Anatomical Sciences, American University of Antigua, College of Medicine, Osbourn, ATG

**Keywords:** mucormycosis, coronavirus disease 2019 (covid-19), diabetes mellitus, rhino-cerebral mucormycosis, rhino-orbito-cerebral mucormycosis

## Abstract

Rhino-orbital cerebral mucormycosis has been commonly seen during the coronavirus disease 2019 (COVID-19) pandemic. Several factors responsible for etiology and pathophysiology have been identified, among which corticosteroids and diabetes have contributed to the lion’s share of the outbreak of mucormycosis. In this report, we discuss a case of a 41-year-old non-diabetic male with a recent convalescence from COVID-19 infection presented with gradual vision loss and loss of sensations in his right eye. He was found to have periorbital swelling, restriction of extraocular movements in all gazes, chemosis, ptosis of the right eye, and right maxillary sinus tenderness. His serum investigations, radiologic findings, and blood culture were indicative of rhino-orbital cerebral mucormycosis. He was started on systemic liposomal amphotericin B immediately and underwent aggressive surgical debridement. A high index of clinical suspicion, aggressive multifaceted management, and follow-up are needed to have successful outcomes, thereby lowering the morbidity of coronavirus-associated mucormycosis.

## Introduction

Coronavirus disease 2019 (COVID-19) has been associated with a wide range of bacterial and fungal co-infections and superinfections. COVID-19 symptoms vary widely, ranging from simple upper respiratory infections to fatal complications [1]. While associated with COVID-19, mucormycosis is a potentially fatal infection causing high mortality and morbidity rates [2]. Mucormycosis is a ubiquitous fungus and is observed to cause blood vessel thrombosis and necrosis of the surrounding tissue due to its angio-invasive properties and associated complications such as cavernous sinus thrombosis, osteomyelitis, and even death [2,3]. We report a case of a non-diabetic 41-year-old male patient who developed invasive rhino-orbital cerebral mucormycosis during his recovery from COVID-19 infection.

## Case presentation

A 41-year-old male with a recent convalescence from COVID-19 infection presented with complaints of vision loss in the right eye associated with headache and nasal congestion for the last 15 days. Loss of vision in the right eye was gradual in onset and progressive in nature and was preceded by intermittent episodes of headache and nasal congestion over the last month. His history revealed a recent recovery from COVID-19 infection after a course of dexamethasone and hydrocortisone for 28 days, along with symptomatic management. He had no history of similar episodes in the past. His past medical history was non-significant, and he stopped smoking five years ago.

**Figure 1:**
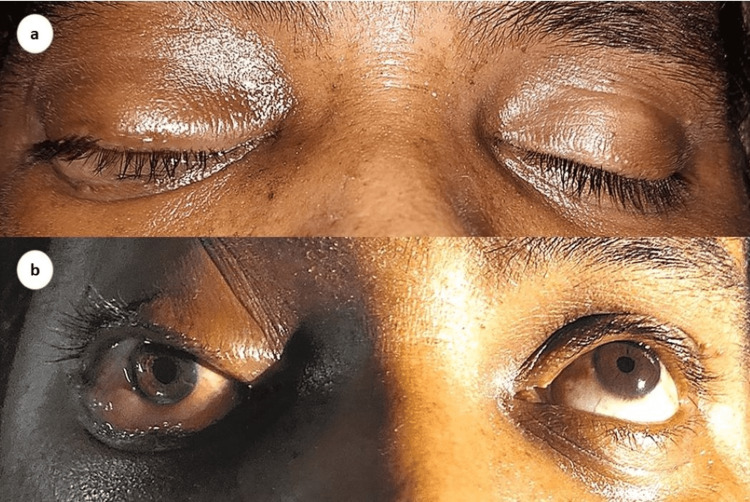
Right eye showing eyelid edema (a), chemosis with fixed and dilated pupil (b).

**Figure 2:**
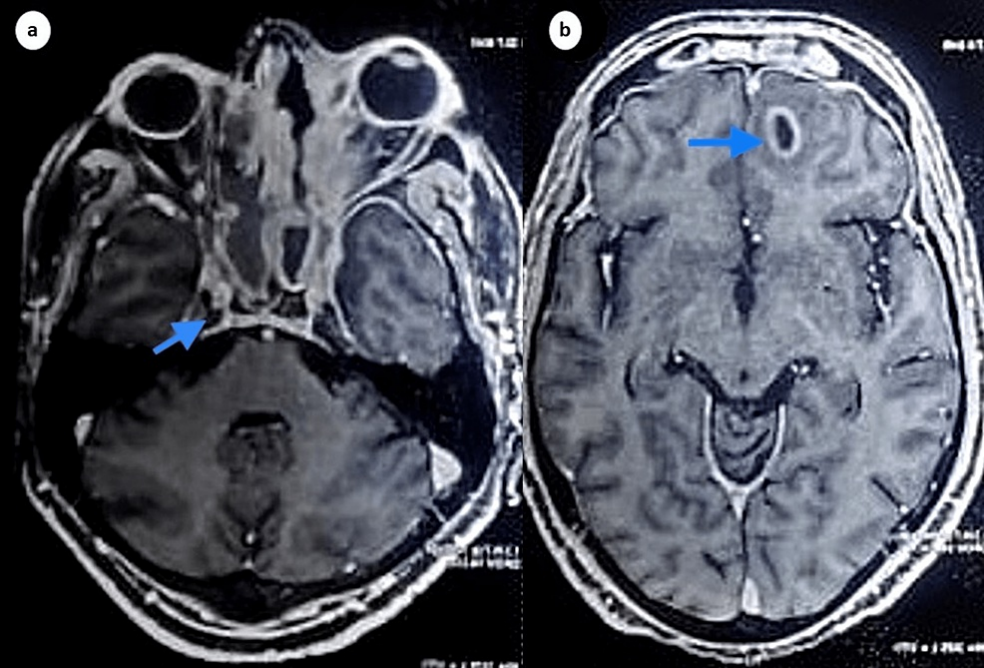
MRI brain demonstrating heterogenous enhancement of right cavernous sinus (a) and cerebral abscess in the right frontoparietal region (b).

On initial evaluation, he was alert and oriented to person, place, and time. He was vitally stable and had a mild fever (100°F). On physical examination, there was eyelid edema, mid-dilated and fixed pupil, proptosis, chemosis, and restriction of extraocular movements of the right eye in all directions (Figure 1). His hematological and biochemical parameters were unremarkable except for leukocytosis (Table 1). His viral workup for human immunodeficiency virus (HIV), hepatitis B, and C were negative. Blood culture and sensitivity results were positive for right-angled non-septate hyphae indicative of mucormycosis. The patient underwent magnetic resonance imaging (MRI) which showed heterogeneous enhancement of the right cavernous sinus, indicative of inflammation, with evidence of necrotic areas and cerebral abscess in the right frontoparietal region, indicative of cerebral mucormycosis (Figure 2).

**Table 1:** The result of initial laboratory tests.

Parameter	Lab value	Reference range
Hemoglobin	12.9	12-16.5 g/dL
Red cell count	4.1	4.2-5.5 million cells/uL
White cell count	14300	4000-11000 cells/uL
Platelet count	190,000	150,000-450,000 cells/uL
Serum creatine	1.1	0.7-1.3 mg/dL
Blood urea nitrogen	35	6-35 mg/dL
Alkaline phosphatase	198	< 240 IU/L
Alanine aminotransferase	49	< 40 IU/L
Serum sodium	138	135-145 mmol/L
Serum potassium	3.9	3.6-5.2 mmol/L
Serum calcium	8.2	8.6-10.3 mg/dL
Fasting blood sugar	86	< 99 mg/dL
Hemoglobin A1c	5.7%	< 5.7%
Erythrocyte sedimentation rate	21	< 12 mm/hr

His laboratory, radiographic, and microbiological evaluation resulted in the provisional diagnosis of pansinusitis with mucormycosis and right orbital cellulitis. He was started on systemic liposomal amphotericin B 10 mg/kg daily and was planned for aggressive surgical debridement with lid-sparing orbital exenteration of the right eye and endoscopic debridement of the right sinuses to prevent the spread of the infection to the cerebral veins.

Histopathology of the lesion confirmed the presence of right-angled non-septate hyphae and was diagnosed with rhino-orbital cerebral mucormycosis. The swelling subsided, and symptoms gradually improved. Postoperative MRI of the brain and orbits showed residual sinus disease features, right orbital, and intracranial extension with a fungal abscess resolving in the right frontal region. He was continued on amphotericin B for the next four weeks, and the patient was monitored for drug compliance and possible side effects at regular visits. He was discharged after otolaryngology and neurology consultation.

## Discussion

Although mucormycosis exhibits a wide spectrum of clinical syndromes, one of the most devastating manifestations would be rhino-orbital cerebral mucormycosis [4]. This rare, opportunistic infection causes pathologic symptoms in the sinus, orbit, and cerebrum and is primarily caused by the *Rhizopus oryzae* [5]. It is associated with a wide range of ophthalmological clinical symptoms where proptosis and ophthalmoplegia are two of the most common presenting complaints [6]. Other ophthalmic signs may include periorbital edema, eyelid gangrene, cellulitis, ptosis, orbital apex syndrome, and cavernous sinus thrombosis [6,7]. Major risk factors for mucormycosis are type II diabetes mellitus (particularly with diabetic ketoacidosis), HIV infection, hematological malignancies, lymphopenia, neutropenia, hemochromatosis, deferoxamine therapy, immunosuppressant use, chronic inflammatory diseases, and prolonged steroid use [7,8].

Mucormycosis is highly resistant to most antifungal drugs; however, amphotericin B, posaconazole, and isavuconazole have been significantly effective in managing mucormycosis [9,10]. Mucormycosis, also called “black fungus,” is a fungal infection that continuously affects COVID-19 patients and has become an imminent danger in some countries already experiencing a COVID-19 crisis. Naruka et al. highlighted 79 cases of confirmed COVID-associated rhino-orbital mucormycosis. In this study, 67 patients had a positive history of diabetes, and 12 had steroid-induced hyperglycemia. However, all the patients were hyperglycemic during the mucormycosis diagnosis [11]. Baskar et al. reported a case of rhino-orbital cerebral mucormycosis caused by COVID-19 infection in a non-diabetic patient [12]. Hannan et al. also reported a case of COVID-19-induced mucormycosis in a non-diabetic patient diagnosed with non-Hodgkin lymphoma [13]. Mucormycosis is usually reported in patients with compromised immunity with associated comorbidities. The most common site of infection is the head and neck, which typically constitutes 50% of all cases [6]. The spread of the infection can lead to rhino-cerebral infections such as rhino-orbital-cerebral mycosis, a medical emergency where early intervention is necessary. The most common etiology for such emergencies is believed to be *R. oryzae* [7].

Important interventions for controlling mucormycosis are early diagnosis, reversing the predisposing factors, surgical debridement of the infected tissue, and relevant therapeutic antifungal therapy [13]. COVID-19 patients have been prescribed steroids, which may suppress immunity in recovering and recovered COVID-19 patients and thereby can trigger mucormycosis infection [14]. The patient described in this case was treated with steroids for 28 days. He also had a history of recurrent right eye abscesses, which were treated conservatively and never with antibiotics and surgical drainage performed. This could have predisposed the disrupted skin to become a reservoir of pathologic fungi not limited to mucormycosis. We can also imply that his vision loss was caused by a thrombus that occluded his central retinal artery caused by the mucor infection. Coronavirus damages blood vessels and airway tissues leading to a higher fungal burden in patients with diabetes mellitus or other lymphopenic comorbidities. The standard treatment is surgical debridement to prevent the spread of infection to blood vessels in the brain and antifungal therapy to avoid the typical hematological systemic spread of the fungus [15]. We suspect the patient described in this case report had blood vessel and lung tissue damage from the COVID-19 infection and a weakened immune system due to the corticosteroid treatment [16]. These risk factors facilitated a fungal infection. However, due to his previous history of recurrent right eyelid abscesses and his MRI results, we cannot rule out a previously undiagnosed mucormycosis infection exacerbated by COVID-19.

## Conclusions

Early diagnosis and prevention of the spread of mucormycosis are crucial because of its high mortality and morbidity. COVID-19-associated mucormycosis is managed by a multidisciplinary team to improve the outcome of the disease. Using corticosteroids judiciously, having access to expert evaluation to diagnose the infection, and increasing awareness are important aspects of controlling the infection. In addition, antifungal treatment and timely surgery are keys to higher survival rates in patients diagnosed with mucormycosis.
